# Chronobiological Analysis of Blood Pressure in a Patient with Atrial Fibrillation at the Development of Heart Failure and Its Therapeutic and Surgical Treatment

**DOI:** 10.1155/2013/490705

**Published:** 2013-02-27

**Authors:** Sergey Chibisov, George Katinas, Inna Brodskaya, Aleksandr Ertman, Grigory Gromyko, Aleksandra Konradi, Oleg Mamontov, Anna Merkuryeva, Ekaterina Polunicheva, Evgeny Shlyakhto, Anna Soboleva, Sergey Yashin, Bharadva Bhavdip

**Affiliations:** ^1^People's Friendship University of Russia, Moscow 117198, Russia; ^2^Saint Petersburg State Medical University, St. Petersburg 197098, Russia; ^3^VA Almasov Heart, Blood and Endocrinology Centre, Russia

## Abstract

Dynamics of blood pressure (BP) and heart rate (HR) was traced by automatic monitoring every 30 min uninterruptedly along several months in a patient suffering from combined atrial fibrillation and heart failure during the development of disease and its therapeutic and surgical treatment (pacemaker implanting and atrioventricular ablation). Analyses of spectral components as well as signal's shape revealed instabilities in circadian and semicircadian parameters. A new approach for signal's form description without using cosine approximation is suggested. The meaning that referring a patient as dipper, night peaker, or nondipper might be useful at choosing tactics of his treatment is impugned, because all these “types” can transform themselves in the same person in few days. Optimization timing of treatment provides better results if not the “types” of daily profile would be taken to account but the real form of the BP-signal and timing its first and second derivatives.

## 1. Introduction

The peculiarity of the case described in the paper was a possibility to trace objectively and in many details the hemodynamic changes continuously at all stages of the development and treatment of the heart disease: from the very beginning of cardiac insufficiency to its culmination, next therapeutic correction and final surgical intervention.

Uninterrupted monitoring of systolic and diastolic blood pressure (SBP and DBP) and heart rate (HR) provided recording data, and next applying of specialized chronobiological programs ensured obtaining quantitative information dealing with dynamics of the process. 

Before the events described, the patient (GSK, further the patient, P, a man of 82), was suffering for years with essential hypertension. Atrial fibrillation with AV connections dysfunction was registered during the last 6 years, and he had renal failure during the last 3 years.

Cardiovascular functions (SBP, DBP, and HR) are monitored in P uninterruptedly since 1998 (the second continuous monitoring in the world). Every 30 minutes data are automatically registered by TM-2421 recorder (A & D, Japan) and after their cumulation transferred into the computer. 

Dynamics of processes was analyzed using specialized software for analyzing trends, global, and gliding spectra [1] and the signal's waveform [2, 3], methods previously described shortly in [4]. For 14 years it provided an opportunity to observe the behavior of cardiovascular functions at many very different changes in environmental conditions and external impacts to the organism [5–8].

## 2. Short History of the Disease

Hypertension was first diagnosed in P in 1959. Its passing was favorable; the subject until 1988 regularly participated in sports with high physical loads and was well tolerated to oxygen deficiency (mountaineering up to 5000 m above the sea level). Manifestations of heart failure were never noted.

In 2005-2006 paroxysms of atrial fibrillation had appeared, with periods of bradycardia when the HR was less than 40 bpm. In process of time the frequencies of paroxysms arise more often. Nevertheless their ECG registering up to 2006 was not done (objectively their presence could be confirmed indirectly by changes of the oscillometric signal's waveforms registered by the recorder during monitoring).

In 2007 appendectomy was implemented using general anesthesia, and in about a week acute renal failure developed. P was turned to the nephrological clinic. During the treatment span the blood pressure often was elevating up to 190 (SBP) and 120 mmHg (DBP). For the first time atrial fibrillation was identified by means of ECG. After 2 months of treatment P was discharged with a diagnosis of chronic kidney disease, stage II. Later the blood pressure was gradually decreasing; regular careful monitoring and periodic adjustment of medication facilitated this process. Nevertheless atrial fibrillation had passed into a permanent form.

## 3. Methods of Data Analysis

Moving spectra of the whole series for each of variables were computed, as well as the total spectra [2] for different spans of development of the process. Dynamics of the mean value (MESOR), amplitude, and acrophase in the range of 6 to 96 hours were traced. Absence of oscillations (amplitude equal to zero) was accepted as the null hypothesis. The spectral components having probability of the null hypothesis *P* < 0.05 were considered as statistically significant (further for brevity significant). For a more detailed assessment of the circadian signals their real shape (daily profile) was approximated and positions of peak and trough and their quantitative estimation with confidence limits were calculated [2, 3].

The applied methods of analysis allow detecting useful signals among noise and considering the dynamics of parameters as a continuous oscillatory process considering oscillations occurring at different frequencies.

## 4. Results

**Figure 1:**
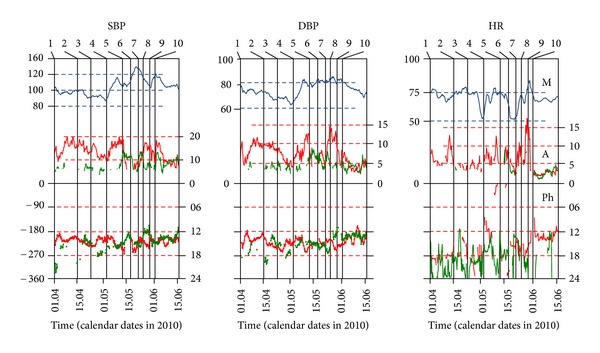
24- and 12-hour rhythms of cardiovascular functions during heart failure development and treatment. SBP: systolic blood pressure, DBP: diastolic blood pressure, HR: heart rate. Blue curves: daily mean values, red: 24-hour rhythm parameters, and green: 12-hour rhythm parameters. Rhythm parameters: M: mesor, A: amplitude, and Ph: acrophase. Abscissae: above: stages of development (according Table 1), below: calendar time (dates in 2010). Ordinates: upper row left: BP or HR values (mmHg, beats/min), upper row right: amplitudes (same units of measurements), lower row left: acrophases (degrees of cycle), and lower row right: acrophases of 24-hour rhythm (clock time).

**Figure 2:**
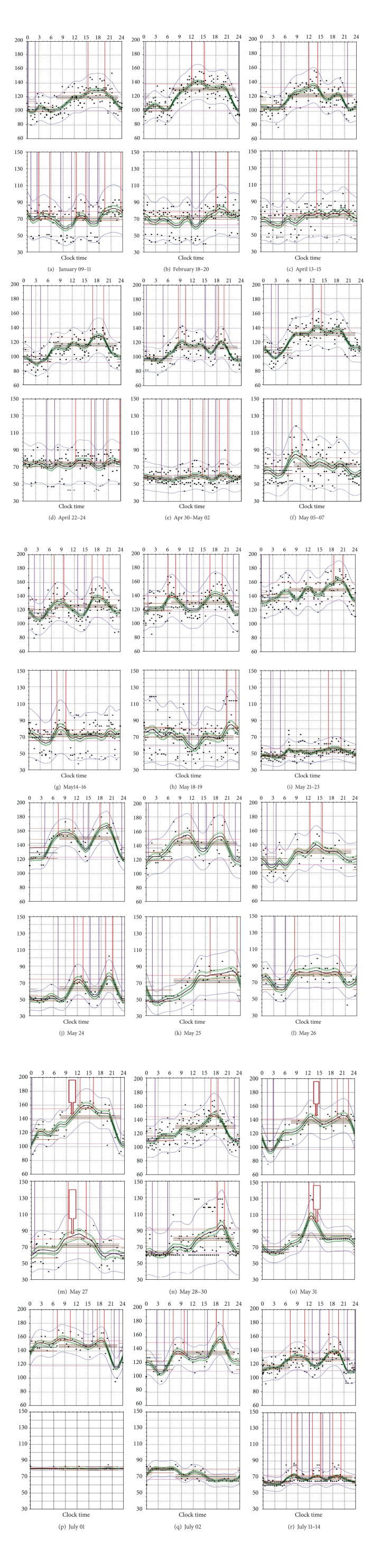
Daily profiles of systolic blood pressure (SBP), and heart rate (HR) before heart failure and during its treatment. ((a)–(r)): Subsequent stages of development. Upper row: SBP; lower row: HR. Abscissa: clock time; ordinates: variable's value (SBP: mmHg; HR: beats/min). Black dots: separate records, Black curve: approximation of the process, Green lines: 95% confidence limits (CL) of approximation, blue lines: 95%CL of records population, Horizontal brown lines: middle night-time and middle day-time variable levels and Vertical red lines: positions of top and statistically significant elevations during the 24-hour span (99%CL-s). Vertical violet lines: positions of bottom and statistically significant decreases during the 24-hour span and their (99%CL-s).

The main quantitative characteristics of 24- and 12-hour rhythmic spectral components are presented in Tables 1, 2, and 3 (the higher harmonics of the circadian rhythm were not significant) and in Figure 1. Changes in daily profiles of SBP and HR are shown in Figure 2.

### 4.1. State before the Acute Disease

During the first quarter of 2010 subjectively the state of the P remained satisfactory. Hemodynamic parameters at this time are shown in Figure 1 (segment 1-2).

All this time P took in beta blockers, calcium channel blockers, and ACE inhibitors. Every few days, the monitor records were analyzed, and dosage, and timing of antihypertensive medications were regularly corrected in accordance to their changes. As a result of such adjustment rates of BP exceeded the commonly accepted target values only rarely (10% of all measurements); see Figures 2(a)–2(c).

The daily mean SBP value changed slightly, rising from 114–121 in January to 118–126mmHg, in the middle of February, and then dropped again to 112–116mmHg in the middle of April (expression of the seasonal changes?).

The amplitude of the 24-hour rhythm component (A-24) at 10–20 mm. 12-hour component (A-12) was expressed rarely, and when it was statistically significant, its amplitude varied from 5 to 10 mmHg. Presence of this ultradian component was manifested by additional waves of the daily profile (see Figures 2(b)-2(c)).

24-hour rhythm acrophase (AF-24) fluctuated around 225° in the range from −187 up to 262°, that is, about 15 hours (from 12 : 30 to 17 : 30). AF-12 (when this rhythmic component was significant) varied around 240° (−30 to −360°).

Dynamics of DBP repeated in the main features the dynamics of SBP. Peaks and troughs of DBP were coinciding, and dynamics of daily mean values, amplitude of oscillation and their acrophases were similar (synchronous). 

The daily mean level of HR ranged around 75 bpm, its A-24 ranged from 5 to 12, and A-12 (when it was statistically significant) was less than 5 bpm. AF-24 was not synchronized with that of systolic and diastolic blood pressure; most often it fell to 18–21 hours. AF-12 was very unstable, varying from −180 to −360°.

### 4.2. Changes in the Profile of Hemodynamic Parameters during Progression of the Disease, after Joining of Heart Failure and at Medication and Surgical Treatment

On April 18, 2010 the patient developed acute respiratory illness that lasted about a week and manifested as a poor health, a runny nose, and eye-watering (see Figure 1, Sections 2-3); the temperature was not overdue 37°. The daily mean of BP becomes decreased if compared with the previous few weeks; A-24 was progressively decreasing, especially in DBP. A-12 has not changed. HR daily mean decreased, but the A-24 and A-12 did not change. In the SBP profile circadian rhythm dominated, and small 6–8-hour variations were superimposed to it (see Figure 2(d)). Circadian rhythm of HR was not regular, and when he nonetheless appeared his A-24 was reduced, and acrophase unstable (see Figures 1 and 2(d)).

On April 26 oedema of feet and ankles appeared. BP was decreasing in combination with low A-24 and bradycardia (see Figure 1, Sections 3-4 and Figure 2(e)). The P appealed to the physician. Following his advice, timing adjustment of regular P was canceled and changed to the standard procedure taking medicine in the morning and in the evening; the preparations were also changed.

On May 5 dyspnoea joined, which occurred even at low physical activity (see Figure 1, Sections 4-5). The level of blood pressure as well as A-24 and A-12 began to grow. BP values became above 140 mmHg in 19% of measurements; sometimes they exceeded 160 mmHg. The mean heart rate returned to normal, but, because of tachycardia affiliation, amplitude of oscillations increased (see Figure 2(f)).

On May 11 diuretics medication began. On May 14 because of progressing heart failure the P was admitted to a therapeutic cardiology clinic. During his stay in the hospital P had the opportunity to continue the continuous BP and HR monitoring but analysis of its results was carried out retrospectively.

After May 14, due to intensive diuretic therapy (both medicamentous and drip feeds), the phenomenon of heart failure has been copped, but BP had not decreased (see Figure 1, Sections 5-6). Circadian rhythm has stopped: A-24 abruptly decreased and ceased to be significant (note to break the red curve, reflecting the A-24 in Figure 1 on the fragments of SBP and DBP). At the same time the 12-hour rhythm became intensified; its amplitude increased more than twice (green curve on the same fragment). HR began to decline, but its A-24 has doubled and A-12 has become more significant. 

As a result, BP has become bimodal with the largest rises in 6–9 and 16–20 and downturns in 0–4 and 11–16 h (see Figures 2(g)-2(h)). 12-hour and even shorter oscillations dominated in HR, combining with apparently circadian profile but with the inverted phase. 

**Figure 3:**
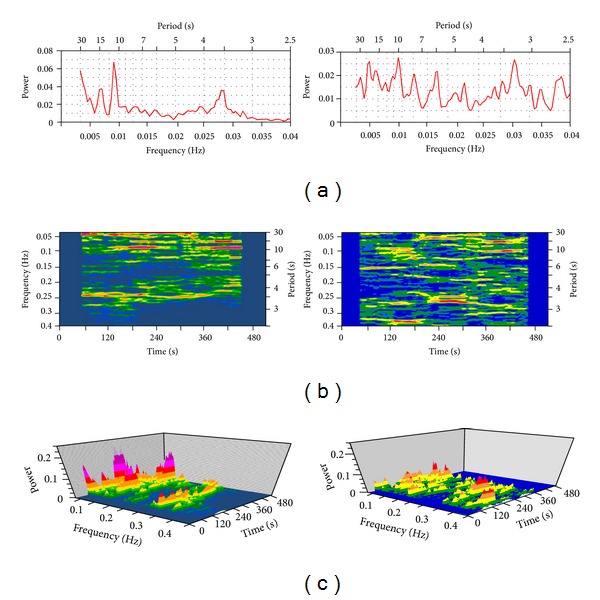
Spectra of electrocardiograms (ECG) in two persons. Left fragment: sinus rhythm in patient B; right one: atrial fibrillation in patient GSK. (a): global spectra of 9 minutes record. Abscissas: spectral components, below: frequency (Hz), and above: period length of oscillations (sec); ordinate: power (*η* ^2^) of spectral components (*η* ^2^ = ratio of described and general variances). ((b) and (c)); 3D gliding spectra of the same records. and (b): view from above (elevation = 90°); (c): view from aside (elevation, rotation, and perspective = 30°). Abscissa: time from starting records (sec), ordinate: frequency (Hz), and applicate: spectral power marked by different colors; blue: statistically not significant values, green components significant at 0.05 > *P* > 0.01, yellow: at 0.01 > *P* > 0.001, orange: at 0.001 > *P* > 0.0001, and red: at 0.0001 > *P* > 0.00001.

During indwelling in the clinic Holter ECG monitoring was carried out, and pauses of more than 2.5 sec were revealed. The necessity of pacemaker implantation become obvious. Retrospective analysis of the ECG spectrum is shown in Figure 3.

To May 19 edema disappeared, shortness of breath decreased, and drop-feed injections were stopped. BP (particularly SBP) remained high exceeding in 69% of the measurements 140 mmHg (see Figure 2(i)). A-24 restored, but does not exceed the A-12 one. Bradycardia restored too (see Figure 1, Sections 6-7). Circadian rhythm of HR heart rate appeared again, but its A-24 was very low (see Figure 2(i)). According to Holter monitoring HR during the day span often happened to be rarer than 40 bpm, and it was considered as a sign for surgical intervention. 

On May 24 the P was transferred to the surgical department. BP remained high, A-24 increased, especially in DBP even surpassing that of the SAD (see Figure 1, Sections 7-8). BP profile was unstable, varying from one day to another, and the ratio of 24- and 12-hour rhythm components varied greatly too (see Figures 2(j)–2(l)). In HR A-24 was predominant, but the phase of the HR rhythm was unstable (see Figures 2(j)–2(l)).

On May 27 the dual chamber pacemaker was implanted (see Figure 1, event 8). SBP began to decline, but the level of DBP did not change. 24-hour component of rhythm clearly dominated; 12-hour one was weak. HR did not fall below 65 bpm; there was a tendency to tachycardia. A-24 increased drastically (see Figure 1, Sections 8-9, and Figures 2(m) and 2(n)). ECG recording demonstrated necessity of interruption of the atrioventricular conduction pathways. Next observation had shown that ventricular tachisystolia could not be copped by usual therapeutic treatment. Cardioversion was not acceptable because of patient's age and heart chambers size.

On May 31 at 1-2 PM atrioventricular node ablation had been performed (see Figure 1, event 9, and Figure 2(o)). The HR level was established by means of pacemaker tuning, but the mean BP remained high (83% of measurements exceeded 140 mmHg). No any daily or ultradian fluctuations of BP were checked after surgery during the next 30 hours (up to the evening of the following day). BP began decreasing only after 9 PM, June 1 (see Figures 2(p)-2(q)).

On June 2 the P was discharged from the hospital (Figure 1, event 10, and Figure 2(q)). The manifestations of arrhythmia did not disturb him anymore. The possibility to make regular analyses of the monitor records and to perform according timing corrections of antihypertensive drugs became renewed.

The BP mean began decreasing and stabilized in 1 week (since June 9). Excesses of SBP over 140 mmHg occurred only in 16% of records. A-24 also declined and stabilized at values which were lower than ones in the beginning of the year. A-12 remained significant and was equal to A-24. Because of it the circadian profile remained bimodal, although bimodality was not as strong as during May 14–20 (see Figure 2(r)). The HR profile was keeping pacemaker's setting—60 bpm at night and not less than 65 during the day time (see Figure 2(r)).

## 5. Discussion

### 5.1. Methods for Detection Rhythms and Estimating Their Parameters

In this paper all computing was made by means of original program complex, elaborated especially for unequidistant data, which are most often used in medicobiological research.

In the standard software elaborated for technical purposes the warranty for precision of results is equidistance of data; the main components of rhythm (24- and 12-hourly) are often evaluated in those programs after their approximation by means of cosine function—the method named “single cosinor” [9]. It is widely used to analyze the results of BP and HR monitoring [10], although in the standard software usually neither cosine parameters nor their statistical significance is estimated.

The first program used during our research (KEKS 2 [2]) permits doing calculations in unequidistant series (having gaps in data). It detects the whole spectrum of periodic oscillations within the user-defined band of frequencies and evaluates mesor, amplitude, and acrophase as well as their statistical significance for every spectral component. A total series is used, thus results of analysis should be called the “global spectrum.”

The second program (GLISSER 3) allows to trace dynamics of spectral components and their parameters in the series and provides revealing their amplitude and frequency modulations [2]. Results of analyses are called “gliding spectrum.” Specialized version of this program (GLIRR) is adapted for the gliding spectral analysis of electrocardiogram and creates 3D visualization of the process [2]. Well-known method of time serial sections [11] is reproduced in GLISSER as one of the possibilities among many others. 

Because all three programs use trigonometric approximation, they smooth the real form of signals too much; they detect periodical signals and reveal their modulations, but too many cosine harmonics should be used for detailed description signal's real shape; in particular, the straight parts of the profiles are reproduced as curved. 

For reproducing those parts of oscillations which allow to judge the true state of peaks and troughs of the process, and the steepness of its ups and downs, and time of their distribution the program “FORM” was elaborated.

Accuracy of shape description is determined by user by means of setting initial parameters. The program FORM [2] provides those calculations. It is based on the modification of Sawitsky-Goley filter and does not use sinusoidal transformations. The program is able to detect in unequidistal and noisy series even such difficult for description signals as having saw shaped or rectangular cycles.

### 5.2. The Behavior of 24- and 12-Hour Oscillators under Extreme Conditions

Results of computing time series by gliding spectral programs might be visualized in 3D graph. Coordinates of its base are frequencies (ordinate) and time (abscissa). Values of spectral parameter (e.g., power or amplitude) are presented. Constant (stationary) rhythm looks in 3D representation like a mountain crest going parallel to abscissa and having everywhere equal heath proportional to the spectrum parameter. If amplitude (or power) is changing in time it reflects in the height of the crest. If frequency of rhythmic oscillations is modulated position of crest is moving along the ordinate axis. When looking at such graph from above (elevation of viewer as 90°) the crest looks likes a stripe.

Different values of parameter might be expressed by different shading or colors (like it is usually made in geographical maps to show relief of the earth surface).

Unlike the serial sections method gliding spectra permit demonstrating the simultaneous development of several spectral components.

**Figure 4:**
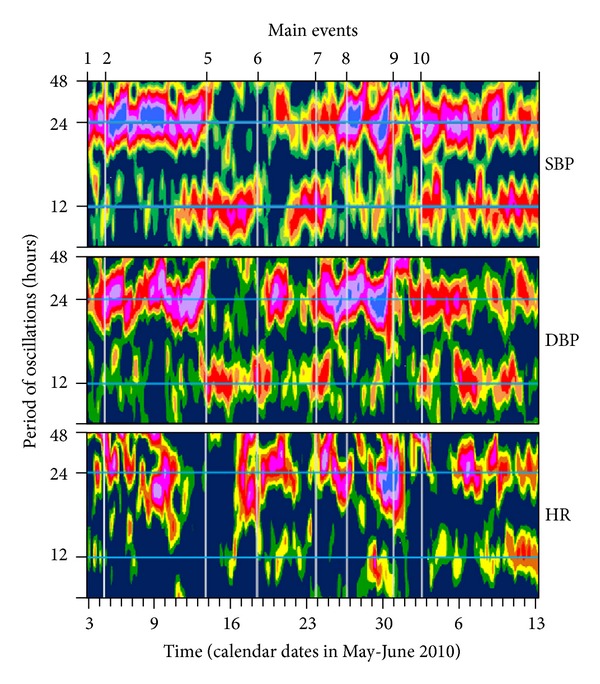
Gliding spectra of systolic and diastolic blood pressure and heart rate during treatment of heart failure and cardiac surgery. Abscissa: time (calendar dates in 2010), ordinates: period length (hours), and applicate: areas corresponding to oscillations of equal power (*η* ^2^); blue: *η* ^2^ < 0.05, green: 0.05 > *η* ^2^ > 0.10, yellow: 0.10 > *η* ^2^ > 0.15, orange: 0.15 > *η* ^2^ > 0.20, red: 0.20 > *η* ^2^ > 0.25, and other colors: *η* ^2^ > 0.25. Main events: 1-2: healthy, 2–5: increasing heart failure, at the therapeutic clinic, 5-6: intensive therapeutic treatment, 6: finish intensive therapeutic treatment, 7: transferring to the surgical clinic, 8: pacemaker implanting, 9: atrium-ventricular ablation, and 10: discharge for home rehabilitation (numbers are the same as in Table 1).

The application of this method demonstrates in Figure 4 the simultaneous development of 24- and 12-hour spectral components. 

24-hour spectral component (24-sc) looks like the band within the boundaries of statistical significance. Interruption of the band means the disappearance of oscillations at this frequency. 12-hour spectral component (12-sc) looks less constant as 24-h one. 

Despite of growing heart failure, the power of 24-sc remained high. Its colors were changing from orange to purple, it means the amplitude of this component was modulated, and the distance between equal colors in the graph demonstrated regularity of modulation with a period of about a week (circaseptan).

At start of observations circadian component was dominating, and the 12-hour one was represented only like small not continuous islands. 

After the heart failure began developing, 12-sc remained weak, but after the start of intensive diuretics medication the 24-sc disappeared, and 12-sc immediately becomes dominant (in daily profile of blood pressure was manifested as bimodality of approximating curve, see Figures 2(f) and 2(g)). After completion of intensive therapy 12-hour component was again lost, and the leading position returned to 24-hour one. Inhibition of 24-sc in the rhythm of blood pressure with the replacement of them by 12-sc has been noted also previously in the same person after a sudden increase in the dosage of antihypertensive drugs [6].

Spectral characteristics of the rhythm occurred changes also under conditions of both surgical procedures, but they were not similar.

Implanting pacemaker on May 27 included several stages: (1) skin incision and entry into the vein, (2) passaging a probe through the veins and penetrating into the heart cavities, (3) fixing electrodes, and (4) removing the probe and suturing the incision. Interrupting atrio-ventricular conduction pathways on May 31 differed fundamentally from that described manipulations mainly at the third stage, when destruction of the anatomical integrity of the organ was performed (which was not done at the first intervention).

The main difference in the rhythms behavior of BP (see Figure 4) was strengthening the 24-sc in the first case but its disappearance in the second one. Its “silence” lasted about 30 hours, after which the oscillations were restored with the same phase relationship as before. 12-sc was depressed immediately after the first operation and reappeared simultaneously with the 24-sc.

Earlier sudden stop of a 24-ch and his subsequent sharp recovery has been observed during rapid transmeridian flights across 9 time zones [12]. Such behavior of the oscillator, from physical point of view, corresponds to the passage by its so-called singular point, when drastic changes of conditions compel oscillations to stop, for choosing new oscillatory parameters adequate to those new conditions. 

First of all, it refers to the phase of fluctuations. They are usually defined by any external synchronizer. During and after flights phase light regimen and social environment routine are inherent for different time zone and serve as pacemakers. 

Moment of ablation was that shock which plunged 24-sc to the singularity, but after surgery lightening and social environment remained the same, thus when a new switch on the oscillator got restored the previous phase of oscillations restored too (see Figure 1). 

The circadian rhythm of HR before the operation was unstable: arrhythmia, immanent for the P; its period length was not strongly equal to 24, but varied within the whole circadian range of 20–28 h; its amplitude and phase were also not constant (see Figure 1) as well as its power (see Figure 4). The first surgery has caused strong increase of the power (see Figure 4), which was manifested as tachycardia (see Figure 2(o)). The following ablation stopped the circadian HR oscillator (as well as in BP), but later the natural circadian variations of HR could not be restored independently and recovered in a week after setting pacemaker which was tuned up to different frequencies for the night and day spans (see Figure 4).

Such behavior of HR rhythmicity supports the previously stated assumption that behavior of 24- and 12-hourly oscillators at various drastic modifications of the organism's activity might be independent [8, 13]. The genes that specify the 24-hour rhythm are now actively studied [14–17]. There are also data that the more frequent (ultradian) oscillations (with periods multiple to 24 hours) might be determined by the combined activity of different 24-hour genes [18–20]. Phenotypically these oscillations are manifested themselves as usually in the activities of peripheral organs [21–23], and their power is increasing during adaptive reorganizations of organism [24, 25]. The observations described in this paper allow assuming that the activity in different peripheral organs has also different anatomical substrates, including specific structures inherent immanently to them.

If our suppositions would be correct we could suggest that the regulatory mechanisms of peripheral circadian and semicircadian heart oscillator include the system of conductive cardiomyocytes. After its mechanical destruction the role of circadian oscillator in relation to the inotropic function is recovered and passes on myocardium of the ventricles, but chronotropic (in terms of circadian and ultradian rhythms) function is not peculiar to ventricles and is lost.

### 5.3. The Physiological Coordination of Diastolic and Systolic Blood Pressure

The natural oscillatory changes in functional activity provide the opportunity of adjustment to changing environmental conditions and, thus, serve as a means for keeping homeostasis: homeostasis should be understood not as a strictly stable state but as coordinated fluctuations not exceeding any limits. These limits, beyond whose pathological changes appear, serve as the boundaries of the norm.

Fluctuations in the various functional systems do not necessarily occur simultaneously (activity of some of them may not coincide) but they occur in concert, that is, in a certain sequence of time; from physical point of view, they are coherent. A lot of external impacts may violate this time coordination (desynchronization occurs). As the most easily diagnosable signs of desynchronized rhythms are changing the correlation coefficients between the processes (which characterizes the tightness of interrelations) and the regression coefficients between them (quantifying the functional dependence of one process from another one) [26, 27]. 

Such an approach would require to assess the mutual coordination of the three registered by monitoring functions—SBP, DAD and HR. However, as the heart rate of the P was erratical due to atrial fibrillation, the analysis was possible only for SBP and DBP.

**Figure 5:**
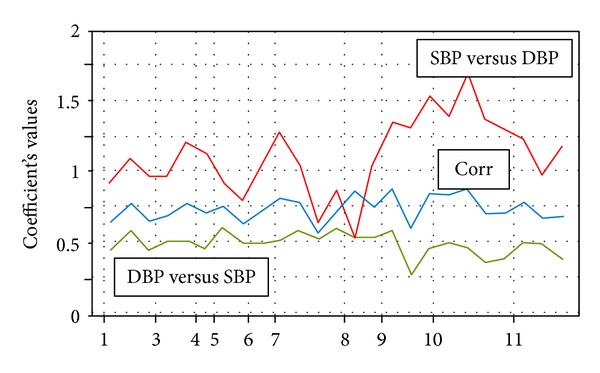
Interdependence of systolic and diastolic blood pressure at different stages of heart failure treatment and cardiac surgery. Abscissa: main events of developing process: 1–3: healthy, 3-4: increasing heart failure, at the therapeutic clinic, 6: finish intensive therapeutic treatment, 7: transferring to the surgical clinic, 8: pacemaker implanting, 9: atrium-ventricular ablation, 10: discharge for home rehabilitation, and 11: after a week at home (numbers are the same as in Table 1). Ordinate: regression coefficients, SBP versus DBP (red), DBP versus SBP (green), and correlation coefficient (blue).

Regression coefficients for SBP versus DBP and DBP versus SBP are not equal (Figure 5). According to their changes in the dynamics of development any two processes are possible to assume, each of them is leading being related to the other one: leader's modification entails the more significant changes in regression coefficient (correlation coefficient at the permutation of regression variables does not change).

Figure 5 shows the change in regression coefficients for SBP and DBP SBP DBP, as well as the correlation coefficients at different stages of development and treatment of disease in P. Changes in correlation coefficients most commonly used in the literature to assess the degree of desynchronization [28–30] almost did not deviate from the values observed in P, during the absence of heart failure symptoms. Changes in the regression coefficient for SBP DBP were expressed much more strongly than those of DBP to SBP.

Taking into account that the value of SBP is largely determined by the state of central regulatory level circulatory system, and diastolic blood pressure—the state of arteriolar level—we can conclude that the consistency of responses in the regulation of the cardiovascular system to the changing conditions of the circulation is the last to take a leadership role. An intensive therapeutic treatment of heart failure, which was accompanied by suppression of the circadian rhythm component, probably temporarily modified the baroreflex (see event markers 5 to 6 in Figure 4). Subsequent reaction proceeded wavelike: increased reflex reactions immediately after the end of intensive therapy replaced by of weakening. Implantation of a pacemaker (see the marker events in Figure 4) was just on the background of a weakening. Immediately after the intervention with the baroreceptor reflex abruptly increased, and the reaction of SBP relative to DBP was inadequately grown up to repeat surgery ablation of conductive paths (see the marker events of 9 in Figure 4). Restoration of normal relations between DBP and SBP occurred after the operation gradually over several weeks after discharge of the patient at home.

These observations demonstrate that the correlation coefficient which is widely used to assess desynchronoses is not the best tool for this purpose. Regression coefficients are much more informative, and from the possible combinations of the two of them regression SBP versus DBP is more sensitive.

Changes of the SBP versus DBP correlation coefficients and regression ones in subjects with desynchronoses, arisen as a result of chaotic schedule of shift work and regularity of sleep and rest [31–33], confirm our present observations.

### 5.4. Assessment of the Daily Profile of the Process as a Criterion for Optimal Timing of the Treatment Effects

That fact that the external impacts on the body, applied at different phases of the circadian rhythm, do not entail the same effect, is known for a long time. This phenomenon is universal; it was shown in relation to light signals, diets, physiotherapy, and many other effects [34–38].

Let us demonstrate some examples from our own observations dealing with experimental surgery. The rate of collagen accumulation of the wound area, the rate of development of the capillary bed in granulation tissue during its healing, and agility of rearrangement peritoneal mesothelium after its injury, as well as other reactions in posttraumatic tissue regeneration are varying depending on whether the injury was inflicted in the morning, afternoon, or evening [39–52].

The main regularities in time dependence of medication effectiveness at their admission at different circadian phase (time of the day) were systematized still about 40 years ago [53]. Taking into account those peculiarities at medical practice was named chronotherapy. Many publications are devoted to optimizing the treatment of hypertensive states by choosing time of the day when the drug should be most effective when using smaller doses.

Such works were usually based on one-day monitoring and the ratio of the averages of daily and nocturnal BP values were calculated (the so-called circadian index). In accordance with the result, the patient is defined as a dipper, nondipper, or nigt-peaker, and antihypertensive medication would be recommended for taking in the morning or in the evening [54–66]. The results were very controversial, and chronotherapy of hypertension began causing skepticism [67].

In 2008, a quite different principle of chronotherapy was proposed—not the simple taking into account only the day time and nocturnal rates, and not the approximation of the 24-hour profile of the circadian curve by the rigid sinusoidal functions: Cosinor method which was proposed a few decades ago [9] does not provide restoration of the real circadian profile because it is able to take into account the ultradian components only of a priori settled periods lengths. This approach makes it possible to determine the rate of change of the process (velocity, the first derivative) throughout the whole circadian cycle as well as the distribution of the accelerations (second derivative). Distribution of accelerations, in turn, makes it possible to decide, when regulatory physiological mechanisms might be included into control of the process [68]. According to results revealed, it should be expedient to use the drug not at the time when BP reaches its maximum values, but when the regulatory mechanisms are just beginning to be switched up: to prevent a fire is easier than to extinguish it only after the flame should be broken out.

**Figure 6:**
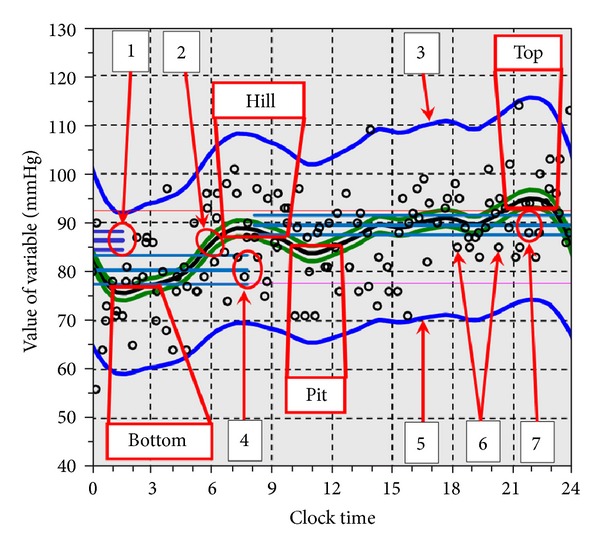
Main features of signal profile. Abscissa: time, ordinate: values of variable. 1: general mean level, 2: approximating curve (profile), 3: upper confidence limit of population, 4: mean level of night values, 5: lower confidence limit of population, 6: separate records, 7: Mean level of night values. Top: highest area of the process, Bottom: lowest area of the process, hill: intermediate elevation, pit: intermediate decrease. Red horizontal bars at those areas: confidence limits of their timing. Hills and tops are shown only if they are statistically significant comparing with the neighboring areas. Ovals include value of parameter and its confidence limits.

Profile of daily course of the process is revealed by means of modified Savitzky and Golay [69] filtering combined with the superposed epochs principle [70]. Revealing profile would be done on the base of three-day monitoring, which makes possible it to calculate all the parameters of the curve with their statistical confidence intervals [3, 71]. Next parameters and their statistical probability might be estimated being appointed by users (Figure 6):

1. distribution of data recorded according to phases of the cycle,
2. average of data values (mean level),
3. approximation of the mid value of the process at various phases of the cycle,
4. peak (top) value,
5. peak's phase,
6. trough (bottom) value,
7. trough's phase,
8. values and positions in the cycle of intermediate elevations and decreases of the profile.

Repetitive investigation of BP and HR circadian profile in the same persons revealed that it does not remain constant; even no acute disease takes place (see Figures 2(a), 2(b), and 2(c)). In all cases, the P looked as a dipper, but the wave shape was quite not equal, and timing of medication for preventing excessive leaps of BP at each case should be different. 

This approach allowed the P to keep his BP for a long time within acceptable limits. After May 4, 2010 chronobiological approach has been canceled, and drugs were taken after traditional pattern—in the morning and in the evening. After that, BP became gradually increasing, exceeded the permissible limits, and returned to the target values only after discharge, when the opportunity of regular analyzing circadian profile and adjusting timing of treatment in accordance with the acceleration of the process had come back (see Figure 2).

Approach based on the described principle has been applied in dozens of cases at treatment hypertensive patients, and has shown good results [72].

Taking into account that the daily profile of BP and HR rarely remains stable for half a week (especially during development of pathology), monitoring for longer than 3-days span may blur the assessment of the actual parameters of the daily profile. From the other side, to settle any medical conclusion based on only one-day recording means to operate in a deficit of information. 

Recommendations made earlier (also with our participation) to carry out continuous monitoring at least one week [73–80] from the point of view of our today experience seem to be informative more scientifically than having actual diagnostic value: the treatment of individual patients is more expedient to carry out for several times three-day ambulatory monitoring at intervals of several days: during treatment of hypertensive states approaching to the target BP values occur gradually, and this mode allows to optimize timing of antihypertensive medications in the better way. 

Our observations impugn the meaning that referring a patient as dipper, night-peaker, or non-dipper may be useful at choosing tactics of his treatment: all these types can transform themselves in few days. From the other side, even if BP “type” was not changed the real peak and trough positions in the cycle might be moved, and such circumstance should require new treatment timing.

Natural mobility of circadian BP profile impugns also the prognostic validity of dipper—nondipper classification. 

## 6. Conclusion

Long-term (multiday) BP and HR monitoring provides valuable information of continuous dynamics of the processes at all stages of their development. This is important, since at the “traditional” planning observation are performed only before any event and after it (e.g., transmeridian flight or surgery), but not during the same event. Unfortunately, today such studies can be carried out only for scientific purposes. If they should be available for every patient, their predictive value for the early detection of cardiovascular diseases becomes very valuable.

Such idea was expressed still many years ago [81–84] and lives up to now, but for most of people it seems to be as an utopia. 

To reach it, progress on several fronts is necessary: (1) miniaturization of recorders in order to rescue patients from troubles associated with wearing the device, (2) wireless transmission of recorded data to any analytical center, (3) establishment of such centers, equipped with the necessary hard- and software, (4) improvement of existing programs for data processing and development of new ones, and (5) training specialists who would be able to use such sophisticated equipment and interpret the results from the medical point of view.

It is difficult to predict how much time will be spent on such work, but if we would like to be ready to use advantages of the latest future technology, the development of theoretical approaches must begin without delay today.

Progress comes much faster than we can imagine. One of the authors of this paper for the first time used the BP monitor still in 1971. It was a large heavy box, its weight was about 20 kg, and it should be carried on the rolling table behind the patient, being connected with the user by wires and an air-tube; records were registered by an ink device; they should be measured and put into computer manually. Let us look at BP monitoring in 40 years.

## Figures and Tables

**Table 1:** Dynamics of the systolic blood pressure in 2010 in the patient GSK.

Stage	Time (calendar dates in 2010)	MESOR	24-hour component			12-hour component		
			Amplitude	Acrophase	*P* value	Amplitude	Acrophase	*P* value
1-2	05/01–19/01	117.5 ± 4.4	14.5 ± 3.2	−241 ± 24	<0.0000	2.08 ± 1.7	−219 ± 200	0.5813
1-2	12/02–26/02	121.8 ± 5.4	19.8 ± 2.0	−226 ± 13	<0.0000	2.02 ± 1.7	−256 ± 154	0.6531
1-2	02/04–16/04	115.9 ± 3.7	15.4 ± 5.2	−208 ± 17	<0.0000	4.2 ± 3.9	−259 ± 115	0.2892
2-3	18/04–25/04	111.4 ± 6.2	14.8 ± 3.3	−226 ± 16	<0.0000	5.9 ± 2.9	−223 ± 31	0.0600
3-4	26/04–04/05	109.8 ± 4.8	11.8 ± 3.2	−215 ± 24	<0.0000	3.9 ± 3.1	−254 ± 37	0.1798
4-5	05/05–13/05	126.7 ± 8.8	16.6 ± 3.4	−222 ± 34	<0.0000	6.5 ± 6.2	−233 ± 19	0.0934
5-6	14/05–18/05	127.4 ± 7.4	5.1 ± 6.8	−237 ± 57	0.2261	11.3 ± 1.6	−209 ± 29	<0.0000
6-7	19/05–23/05	144 ± 6.1	7.2 ± 4.1	−240 ± 31	0.0529	7.2 ± 6.2	−225 ± 33	0.0415
7-8	24/05–26/05	136.1 ± 9.3	13.0 ± 5.2	−213 ± 16	<0.0000	10.1 ± 6.1	−226 ± 25	0.0187
8	27/05	129.4 ± 0.4	16.9 ± 0.4	−216 ± 1	<0.0000	5.9 ± 0.3	−191 ± 13	0.0424
8-9	28/05–30/05	124.3 ± 5.0	14.8 ± 3.9	−231 ± 13	<0.0000	7.8 ± 1.9	−179 ± 20	0.0038
9	31/05	131.4 ± 4.9	12.7 ± 5.9	−210 ± 15	0.0001	5.6 ± 2.7	−180 ± 31	0.0754
9-10	01/06-02/06	136.2 ± 4.3	12.0 ± 7.2	−213 ± 18	0.0001	7.3 ± 5.0	−220 ± 33	0.0211
10-11	03/06–08/06	129.6 ± 9.8	6.5 ± 7.2	−216 15	<0.0000	12.5 ± 6.6	−201 ± 17	0.0035
11-12	09/06–17/06	122.9 ± 3.1	12.4 ± 3.3	−216 25	0.0005	12.5 ± 2.3	−204 ± 20	0.0004

Comments

Stages of developing process:

(1) out of disease (before April 18),

(2) beginning of acute respiratory illness (April 18),

(3) ankle oedema joints (April 26),

(4) dyspnoea joints (May 5),

(5) admission to the therapeutic clinic (May 14),

(6) copping heart failure (May 19),

(7) transferring to the surgical clinic (May 24),

(8) pacemaker transplanted (May 27),

(9) atrio-ventricular ablation performed (May 31),

(10) discharge (June 2),

(11) weekly rehabilitation at home (up to June 9),

(12) stabile state at home (after June 9).

After symbol “±” 95% confidence limits of values are shown.

Patient GSK, a man of 84.

**Table 2:** Dynamics of the diastolic blood pressure in 2010.

Stage	Time (calendar dates in 2010)	Mesor	24-hour component			12-hour component		
			Amplitude	Acrophase	*P* value	Amplitude	Acrophase	*P* value
1-2	05/01–19/01	75.8 ± 2.8	9.1 ± 2.6	−239 ± 30	<0.0000	1.8 ± 1.6	−196 ± 237	0.5044
1-2	12/02–26/02	78.4 ± 3.8	13.7 ± 2.1	−223 ± 22	<0.0000	1.3 ± 1.3	−235 ± 188	0.7178
1-2	02/04–16/04	74.5 ± 2.2	9.3 ± 3.9	−210 ± 26	0.0003	2.9 ± 2.4	−248 ± 158	0.2602
2-3	18/04–25/04	69.6 ± 3.3	9.4 ± 1.7	−237 ± 25	<0.0000	4.2 ± 2.1	−229 ± 73	0.0586
3-4	26/04–04/05	67.6 ± 4.1	6.0 ± 2.4	−229 ± 35	9.44*E*−05	2.9 ± 2.7	−271 ± 70	0.1838
4-5	05/05–13/05	77.4 ± 10.0	9.5 ± 4.5	−225 ± 150	<0.0000	3.9 ± 2.4	−222 ± 26	0.0829
5-6	14/05–18/05	80.4 ± 3.2	3.6 ± 5.8	−150 ± 156	0.3468	7.6 ± 2.0	−225 ± 27	0.0001
6-7	19/05–23/05	82.5 ± 1.8	6.0 ± 5.0	−214 ± 78	0.0847	5.7 ± 2.6	−235 ± 26	0.0012
7-8	24/05–26/05	82.8 ± 2.3	11.0 ± 6.3	−213 ± 36	<0.0000	6.4 ± 3.3	−252 ± 67	0.0184
8	27/05	84.7 ± 2.4	13.8 ± 2.8	−213 ± 30	<0.0000	3.3 ± 1.8	−223 ± 55	0.1898
8-9	28/05–30/05	83.5 ± 4.3	11.8 ± 2.4	−242 ± 19	<0.0000	4.6 ± 1.6	−191 ± 34	0.0283
9	31/05	83.5 ± 0.6	8.8 ± 0.7	−236 ± 42	<0.0000	3.4 ± 1.7	−170 ± 39	0.0649
9-10	01/06-02/06	83.7 ± 1.5	6.3 ± 3.4	−232 ± 51	0.0002	3.4 ± 2.4	−197 ± 21	0.0757
10-11	03/06–08/06	79.6 ± 3.6	6.17 ± 2.9	−211 ± 28	<0.0000	4.7 ± 2.1	−200 ± 17	0.0041
11-12	09/06–17/06	74.3 ± 4.4	4.4 ± 2.0	−202 ± 44	0.0027	4.6 ± 2.2	−195 ± 21	0.0019

Comments are the same as in Table 1.

**Table 3:** Dynamics of the heart rate in 2010.

Stage	Time (calendar dates in 2010)	Mesor	24-hour component			12-hour component		
			Amplitude	Acrophase	*P* value	Amplitude	Acrophase	*P* value
1-2	05/01–19/01	71.7 ± 5.0	7.1 ± 3.6	−325 ± 24	0.0073	3.1 ± 2.2	−319 ± 202	0.2962
1-2	12/02–26/02	73.3 ± 3.0	7.3 ± 2.6	−283 ± 31	0.0004	2.4 ± 2.3	−327 ± 175	0.4040
1-2	02/04–16/04	71.1 ± 5.8	6.1 ± 5.0	−270 ± 62	0.0422	2.3 ± 2.1	−331 ± 106	0.4741
2-3	18/04–25/04	74.6 ± 4.2	3.9 ± 2.3	−260 ± 65	0.0918	1.7 ± 1.5	−316 ± 151	0.5135
3-4	26/04–04/05	65.6 ± 16	3.9 ± 4.0	−276 ± 115	0.1508	2.7 ± 2.9	−286 ± 66	0.2693
4-5	05/05–13/05	71.8 ± 7.0	6.8 ± 5.1	−156 ± 221	0.0613	3.2 ± 3.1	−271 ± 102	0.3629
5-6	14/05–18/05	70.5 ± 5.4	5.0 ± 6.7	−208 ± 289	0.2876	4.2 ± 3.7	−268 ± 33	0.2581
6-7	19/05–23/05	54.2 ± 6.6	4.9 ± 3.4	−232 ± 122	0.0294	3.0 ± 2.2	−243 ± 136	0.1125
7-8	24/05–26/05	64.5 ± 13.2	8.0 ± 4.2	−246 ± 34	0.0022	3.1 ± 2.4	−311 ± 60	0.2628
8	27/05	69.9 ± 3.6	5.2 ± 4.2	−234 ± 12	0.0514	2.6 ± 1.9	−311 ± 116	0.4111
8-9	28/05–30/05	70.3 ± 10.4	11.2 ± 10.7	−262 ± 34	0.0079	5.9 ± 8.0	−246 ± 201	0.1206
9	31/05	81.5 ± 3.5	13.4 ± 4.4	−229 ± 10	<0.0000	5.0 ± 2.6	−150 ± 61	0.0780
9-10	01/06-02/06	76.5 ± 8.1	3.9 ± 8.24	−169 ± 160	0.1519	1.9 ± 2.9	−354 ± 228	0.3926
10-11	03/06–08/06	67.3 ± 2.7	2.4 ± 1.2	−198 ± 45	0.0516	2.0 ± 1.4	−254 ± 22	0.0514
11-12	09/06–17/06	67.9 ± 2.4	3.2 ± 1.0	−201 ± 30	0.0008	3.4 ± 1.7	−241 ± 10	0.0008

Comments are the same as in Table 1.
